# Kappa Free Light Chains: Diagnostic and Prognostic Relevance in MS and CIS

**DOI:** 10.1371/journal.pone.0089945

**Published:** 2014-02-25

**Authors:** Stefan Presslauer, Dejan Milosavljevic, Wolfgang Huebl, Silvia Parigger, Gabriele Schneider-Koch, Thomas Bruecke

**Affiliations:** 1 Department of Neurology, Wilhelminenspital, Vienna, Austria; 2 Department of Laboratory Medicine, Wilhelminenspital, Vienna, Austria; Institute Biomedical Research August Pi Sunyer (IDIBAPS) - Hospital Clinic of Barcelona, Spain

## Abstract

**Background:**

Quantification of kappa free light chains (KFLC) in cerebrospinal fluid shows high diagnostic sensitivity in multiple sclerosis and clinically isolated syndrome patients. However, a clearly defined threshold value is still missing and a possible prognostic value of the KFLC levels in these patients remains undefined.

**Methods:**

Results of KFLC quantification in 420 controls were used to set an upper limit of normal KFLC concentration in CSF under different blood-CSF-barrier conditions. Additionally, KFLC values of MS and CIS patients were assessed and results were evaluated with reference to the patients corresponding disease courses.

**Results:**

The calculated upper limit of normal KFLC-concentration covers 98% of these control patients. Using this cut-off, plasma cell activity in CSF can be detected in 97% of MS patients and in 97% of CIS patients. However, there is no evidence that the extent of KFLC elevation provides prognostic value in MS and CIS patients in this study.

**Conclusion:**

KFLC determination should become a first line screen in the diagnostic algorithms of MS and CIS. The extent of elevation of intrathecal KFLC has no prognostic value on the disease course in MS and CIS patients.

## Introduction

The detection of a humoral immune response by CSF analysis supports the diagnostic process in cases of MS and CIS[1]–[3]. In those patients, plasma cells located in the intrathecal space secrete predominantly IgG and the quantitative and qualitative determination of this marker is part of current generally accepted diagnostic criteria of MS [4]. However, quantitative IgG measurement like IgG-index calculation showed low sensitivity and specificity in different cohorts of MS patients [5], [6] and the diagnostic validity of IgG-quantification in MS diagnostics has been questioned [7]. The current gold standard test for the qualitative demonstration of oligoclonal IgG in the CSF is isoelectric focusing (IEF) followed by immunoblotting [8]. This demanding method of oligoclonal bands (OB) determination reached high sensitivities (88–100%) in several studies in MS patients [9]–[11]. However, a recent meta-analysis reported a significantly lower incidence of OB in CIS cohorts with a sensitivity of 68,6% [10].

The humoral immune response involves the production of both, immunoglobulins (Ig) and free light chains (FLC) of which there are two types: kappa free light chains (KFLC) and lambda free light chains (LFLC). So far several studies have demonstrated elevated levels of FLC in the CSF of MS patients, in particular showing elevation of the KFLC [12]–[15]. More recently different threshold values have been established for a KFLC-index [(CSF KFLC/serum KFLC)/(CSF albumin/serum albumin)] with variable diagnostic performances [16], [17].

There is evidence of a relation between a benign prognosis in MS and an absent or non-detectable humoral immune response in CSF. The absence of OB predicted a relatively benign course and less disability in several studies on MS patients [18]–[21] but results could not be reinforced by recent retrospective studies on big cohorts of MS patients [22], [23]. It has been suggested that the presence of OB in CIS cohorts is combined with a shorter conversion time to MS [24], Tintore et al additionally reported a faster disability progression in those patients [25]. However, these findings could not be confirmed by a study on 208 CIS patients [26]. Moreover, two study groups described a correlation of raised KFLC in CSF with disability prognosis in MS cohorts [27], [28]. And Villar et al demonstrated a predictive value of high KFLC levels in CSF concerning conversion to MS [29].

## Methods

This study was approved by the Ethics Committee of the city of Vienna.

Management: Health service of the city of Vienna.

Thomas Klestil Platz 8/2, 1030, Vienna, Austria.

All participants provided a written informed consent to participate in this study. All samples were anonymized as demanded by the local ethic committee.

### Subjects

Between 2001 and 2010, matched serum and CSF samples were consecutively collected from 861 unselected patients who underwent a lumbar puncture. We reviewed the patients records and established the following diagnostic groups: MS group (n = 65 patients (60 relapsing-remitting MS, 5 primary progressive MS)) fulfilling the criteria of dissemination in space and time for diagnosis of MS according to recent criteria [30]. In none of these cases the CSF results were the decisive diagnostic criteria. The CIS group consisted of 69 patients presenting typical clinical symptom(s) and magnetic resonance imaging (MRI) alteration(s) not fulfilling the modified Barkhof criteria [30] and with no proof of a dissemination in time in the disease course or by MRI-controls. All patients with positive OB (n = 139) formed an OB-positive group. Furthermore KFLC-levels of cohorts suffering from meningitis/encephalitis (n = 81) and from definite neuroborreliosis (n = 8) were examined separately.

To evaluate a possible prognostic value of the KFLC index, MS and CIS-patients with follow-up visits at least 3 years after lumbar puncture were selected. All together we could examine three homogeneous subgroups : 29 patients with relapsing-remitting MS receiving immunomodulating therapy (glatirameracetate or interferon beta), 5 patients suffering from primary progressive MS with no specific MS medication and 24 patients who presented a CIS with conversion to MS within the period under review and who received no immunomodulating therapy. The mean period under review was 55 months. The moderate and high elevated KFLC values were grouped using an arbitrary cut-off and the corresponding clinical courses were compared: changes of the Expanded Disability Status Scale (EDSS), number of relapses, time period until conversion from CIS to MS. Moreover, CIS patients showing no MS conversion within period under review were investigated separately (n = 24).

### Threshold Value of KFLC

A further group was established to define a threshold value of KFLC in accordance to the integrity of the blood-CSF-barrier: From the whole collective of 861 patients the following diagnoses were excluded due to possible intrathecal plasma cell activity, possible inflammatory alterations of the blood-CSF-barrier, paraneoplastic FLC-production or blood contamination of the CSF: MS, CIS, neuromyelitis optica, myelitis, neuroborreliosis, neurosyphilis, neurosarcoidosis, (suspected) meningitis, (suspected) encephalitis, Guillain-Barre syndrome (GBS), chronic inflammatory demyelinating polyneuropathy, Miller-Fisher syndrome, multiple myeloma/lymphoma, intracerebral haemorrhagia. The remaining 420 individuals were selected as our control group (included diagnoses: dementia, apoplexia, epilepsia, migraine, hydrocephalus, cancer, tension headache, peripheral facial palsy). Controls presenting an equal blood-CSF barrier function defined by very similar albumin-ratios (albumin concentration in CSF divided by albumin concentration in serum) were grouped to 23 separate subcohorts of 15–20 individuals each. The highest variation of the albumin ratio within one subcohort was +/−2,5×10^−3^. For each subcohort of the control group the mean value and the standard deviation of the corresponding KFLC-quotient (KFLC concentration in CSF divided by KFLC concentration in serum) values were calculated. These results of mean value +3× standard deviation were taken as the upper limit of normal KFLC-quotient of each subcohort and were representing reference points of an upper threshold line.

### Sample Collection and Laboratory Analyses

A cubital vein was punctured and 6 ml blood collected into a gel-containing standard tube (Vacuette tube, Greiner Bio-One, Kremsmünster, Austria) for analysis of immunoglobulins and KFLC. CSF obtained separately was processed within two hours after lumbar puncture or stored at –80°C until further examination.

Serum albumin and immunoglobulin concentrations were quantified using a Cobas Integra 700 (Roche Diagnostics) with reagents supplied by the analyzer’s manufacturer. CSF albumin and immunoglobulin concentrations were determined by nephelometry on a Behring ProSpec (Dade-Behring, Marburg, Germany) using reagents supplied by the analyzer’s manufacturer. The IgG-index was calculated according to the following formula: (CSF IgG/serum IgG)/(CSF albumin/serum albumin). Results were interpreted using the IgG-quotient/albumin-quotient diagram for demonstration of the intrathecally produced IgG-fraction [31]. Detection of oligoclonal IgG banding was realized with isoelectric focussing on agarose gel on a Helena SPIFE 2000 automated electrophoresis system (Helena BioSciences Europe, Sunderland, UK) and subsequent immunoblotting using the Helena IgG IEF Kit (Helena BioSciences). In collaboration with other laboratories our OB detection unit participates in an external quality control for OB with sample evaluation twice a year.

Both CSF and serum KFLC concentrations were analyzed using a Behring ProSpec with the serum free light chain immunoassay (Freelite, The Binding Site, Birmingham, UK) according to the manufacturer’s instructions. When performing 20 replicate tests on polyclonal sera the KFLC assay showed an intra-assay coefficient of variation (CV) of 7,9% at 0,7 mg/l and an inter-assay CV of 8,7% at 14 mg/l [32].

To evaluate the reliability of the assay for the low concentrations in CSF another intra-assay test with 20 replications was performed by our study group including concentrations of 0,1 mg/l, 0,2 mg/l, 0,4 mg/l and 0,8 mg/l.

Results were expressed as KFLC-index determined by the following ratio: (CSF KFLC/serum KFLC)/(CSF albumin/serum albumin).

### Statistical Analysis

Comparisons between groups were assessed using non-parametric methods (the Kruskal-Wallis test and the Mann-Whitney U test). A two-sided p-value less than 0,05 was considered to indicate statistical difference. The borderline of the KFLC-quotient/Albumin-quotient diagram was constructed using linear regression analysis by Microsoft Excel.

## Results

The intra-assay tests showed low variations when measuring the minor concentrations of KFLC that were regularly found in CSF, with CVs of 4,0% for 0,1 mg/l, 3,4% for 0,2 mg/l, 3,7% for 0,4 mg/l and 2,9% for 0,8 mg/l.

**Figure 1:**
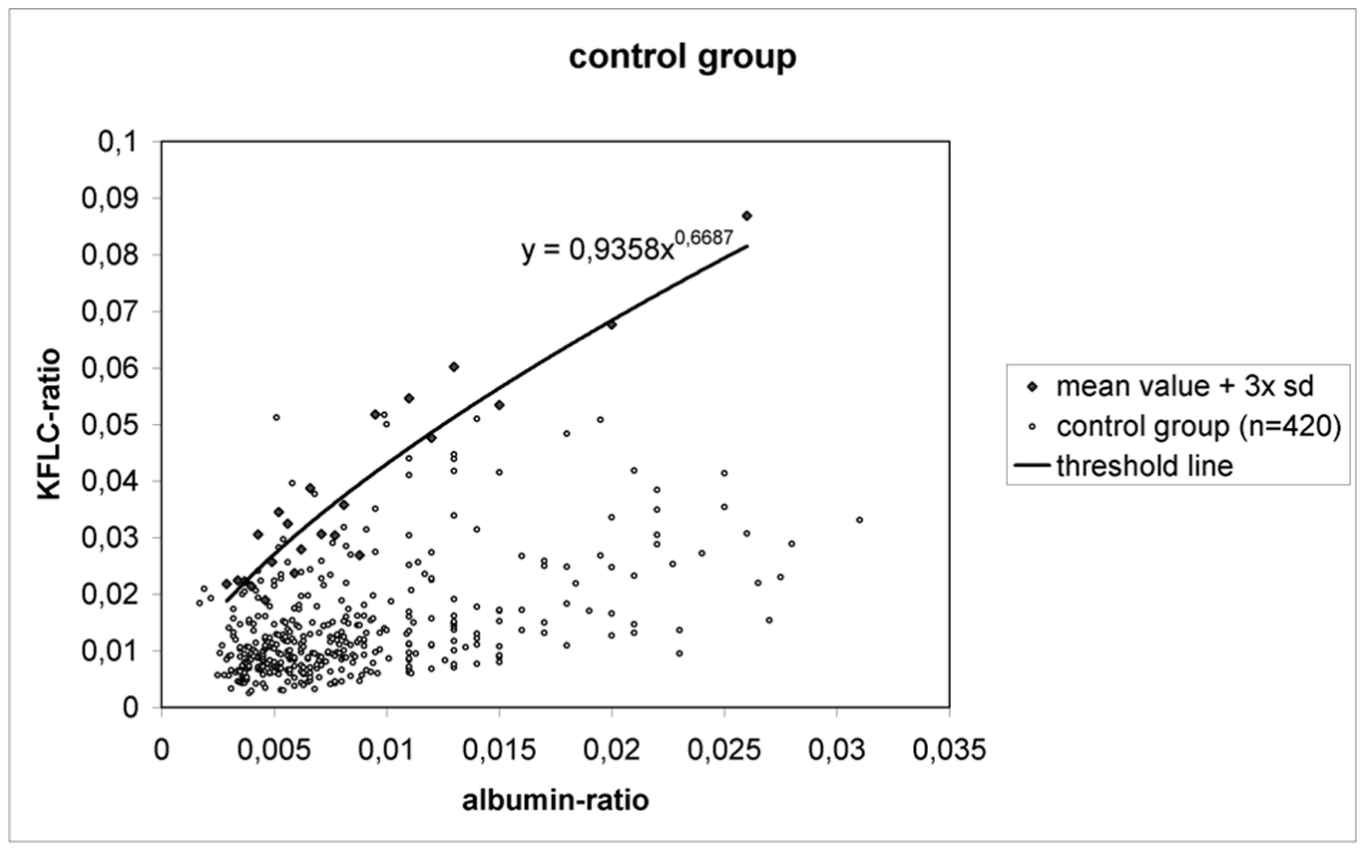
Threshold line of KFLC; sd = standard deviation.

In our control group (n = 420), we found low levels of KFLC in CSF, the mean value was 0,17 mg/l, the mean KFLC-index was 2,12. The mean albumin-ratios of the 23 different subcohorts of our control group covered a range from 3×10^−3^ to 26×10^−3^. The threshold line constructed by using the mean value of KFLC-ratio (KFLC in CSF divided by KFLC in serum) +3× standard deviation of each subcohort covered 412 patients (98%) of our control group (see Fig. 1). The 8 controls presenting with an elevated KFLC-index had the following diagnoses : peripheral facial palsy (2 patients), breast cancer (2 patients, one of them with known cerebral metastasis), lung cancer (1), transient ischemic attack (1 patient), Alzheimer’s disease (1 patient), suspected amyotrophic lateral sclerosis (1 patient).

The formula of this threshold line is:

y = 0,9358 ^x^ x ^0,6687^ (x = albumin-ratio; y = KFLC-ratio ).

**Figure 2:**
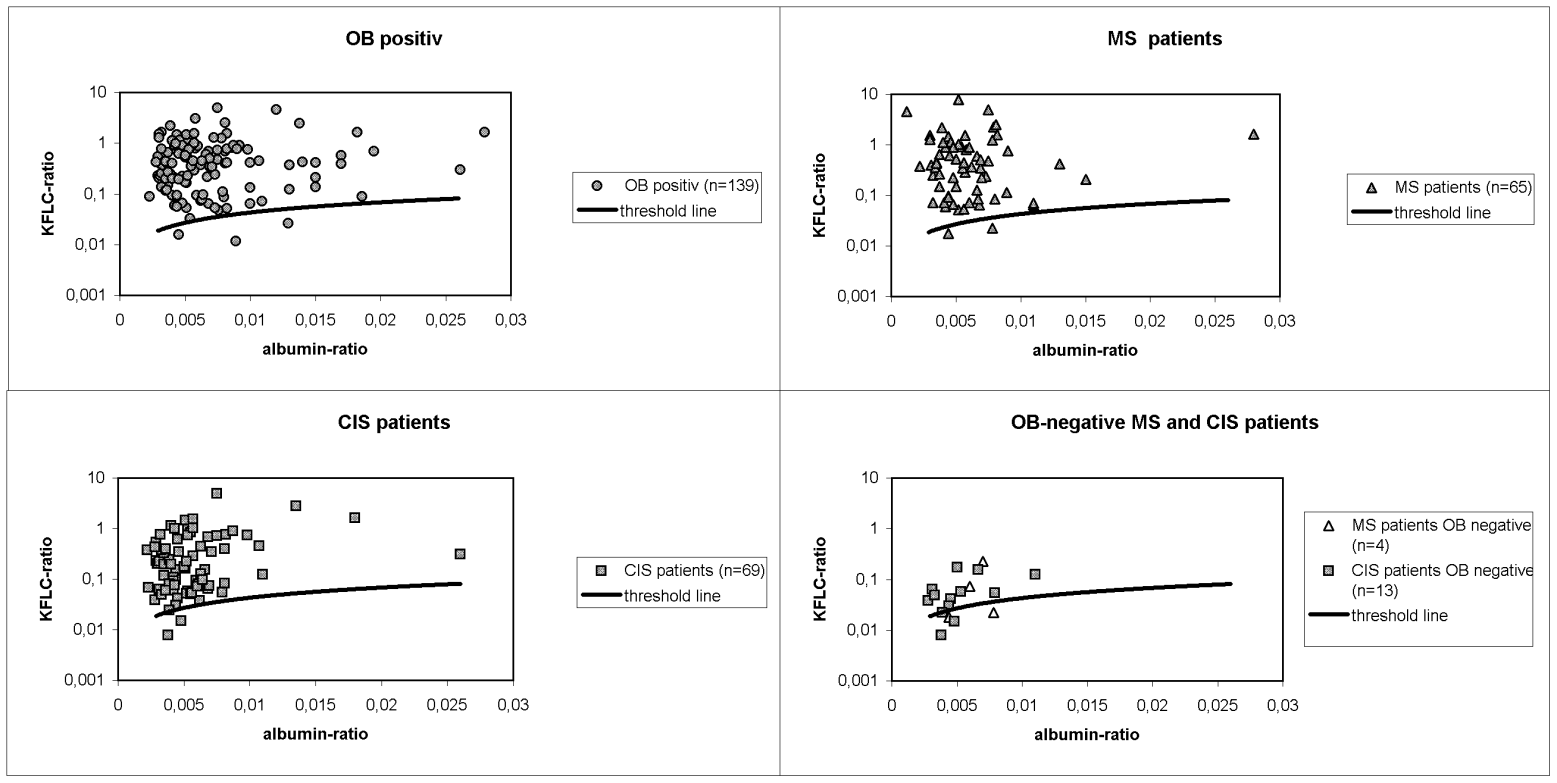
KFLC threshold line in half-logarithmic diagrams with results of different patient groups.

The OB positive group consisted of 139 patients suffering from MS (n = 61), CIS (n = 52), lyme neuroborreliosis (n = 8), meningitis (n = 4), meningoencephalitis (n = 2), neurotuberculosis (n = 2), GBS (n = 2), neurosyphilis (n = 2), subacute sclerosing panencephalitis (n = 1), breast cancer with meningeal carcinomatosis (n = 1), chronic inflammatory demyelinating polyneuropathy (n = 1), paraneoplastic syndrome (n = 1), hydromyelia (n = 1), hydrocephalus internus (n = 1). 78% of this group showed elevated intrathecal IgG levels. When KFLC values of this group were interpreted using our new established threshold line 136 of 139 patients (98%) exhibited elevated KFLC (see Fig. 2). Only three patients presented with positive OB and KFLC values within the normal ranges. The three corresponding diagnoses were one case of hydromyelia, one case of paraneoplastic syndrome and one case of bacterial meningitis.

In the MS group 61 of 65 patients had qualitatively detectable intrathecal IgG production exhibiting OB with a sensitivity of 94%. The IgG-index calculation detected intrathecal plasma cell activity in 85% of our MS group. Regarding our KFLC-index threshold line, 63 of 65 patients (sensitivity of 97%) exhibited an intrathecal humoral immune response in the form of an elevated KFLC-index (see Fig. 2). In 2 MS patients none of our CSF analyses could detect plasma cell activity. All patients with primary progressive MS (n = 5) were detected by all 3 methods (IgG-index, OB, KFLC). The mean KFLC-index in MS patients was 140,74.

CSF and serum samples of 69 CIS patients were examined. 56 of these patients showed OB (sensitivity 81%), 51 presented an elevated IgG-index (sensitivity 74%). Including the KFLC values of our CIS group in the diagram, we could detect intrathecal inflammation in 67 of 69 CIS patients (sensitivity of 97%) (see Fig. 2). In 2 CIS patients none of our CSF analyses could detect plasma cell activity. The mean KFLC-index of the CIS group was 82,37. It was significantly lower compared to our MS group. Both, the KFLC-index values of our MS group and of our CIS group were significantly higher compared to our control group.

Regarding KFLC values of MS (n = 4) and CIS (n = 13) patients with no detectable OB we find several results above or near our constructed borderline (see Fig. 2). All together two MS patients and 11 CIS patients of this subgroup exhibited elevated KFLC-index values. The mean KFLC-index was 13,2. No MS patient but two CIS patients of this subgroup presented an elevated IgG-index.

Patients suffering from meningitis/encephalitis (n = 81) showed a high variation in intrathecal KFLC values. The mean absolute value was 0,89 mg/l (0,14–17,4 mg/l), the mean KFLC-index was 6,51. 59 out of 81 patients had normal KFLC-index values according to our borderline.

All 8 patients presenting with a definite neuroborreliosis showed positive OB and an elevated cell count in CSF and in all of them raised CSF KFLC levels (mean 5,4 mg/l) and raised KFLC-indices (mean 34,1) were discovered.

### Prognostic Value

We formed similar sized cohorts of MS and CIS patients with moderately and highly elevated KFLC-index values and compared clinical changes over time. There was no significant difference between the mean value of KFLC-index of the cohorts with available clinical data and the mean value of the whole MS and CIS groups. Within the period under review there was no evidence of a prognostic value of the extent of KFLC-index elevation concerning turnover-rate in untreated CIS patients and the relapse-rate or EDSS progression in MS patients receiving immunomodifying therapy (see table 1). Moreover, there was no significant difference between the KFLC-index of the CIS group showing MS conversion (KFLC-index 77,8) compared to the stable CIS group (KFLC-index 82,7).

**Table 1:** Prognostic value of KFLC-index in MS and CIS patients.

Relapsing-remittingMS patients(n = 29)	KI>100	KI<100	Mean ageat onset(years)	Immunomodifyingtreatment	MeanKI-value	Mean KFLC-Concentrationin CSF (mg/l)	totalperiodunderreview	meanrelapses/year	p-value(relapses/year)	mean EDSS3 yearsafter onset	p-value (EDSSafter 3 years)
	n = 16	-	27,2	yes	170,3	8,24	63,5 years	0,80	0,42	1,3	0,61
	–	n = 13	30	yes	39,6	2,62	61,5 years	0,59		1,4	
Primary progressive MS patients (n = 5)	KI>100	KI<100						mean EDSS-progression/year			
	n = 2	–	54,5	no	307	25,62	12 years	0,5/year		cohorts too small for statistical analysis	
	–	n = 3	42	no	30,3	2,13	14,5 years	0,55/year			
CIS patients withconversion toMS (n = 24)	KI>50	KI<50						mean time until conversion		p-value (time until conversion)	
	n = 10	–	30,2	no	104	4,68	52 years	6,8 months		0,49	
	–	n = 14	33,4	no	29,8	1,49	58,5 years	8,1 months			

KI = KFLC-index; p-value less than 0,05 is considered to indicate statistical difference.

## Discussion

The importance and use of CSF examination in the diagnostic process of MS seems to have decreased when considering the latest revision to diagnostic criteria of MS [33]. The method is not even mentioned when experts try to formulate a diagnostic algorithm for the CIS [34] However, neither clinical examinations nor MRI analyses can deliver strong evidence of the inflammatory origin of focal clinical deficits and/or MRI alterations. Moreover CSF analyses are essential to rule out other neurological diseases that can mimic MS or CIS [34].

Intrathecal plasma cell activity can be demonstrated qualitatively by IEF but the detection limit of this method considered as the quantity of oligoclonal IgG that needs to accumulate for visible bands is unknown. The diagnostic value can be raised by specialists using a high sensitivity method for OCB detection [11]. The results presented here of CSF examinations from OB negative MS and CIS patients with sizable elevated KFLC-index values give evidence that in 13 patients KFLC determination can detect very low plasma cell activity in the CSF, beyond the sensitivity of IEF calculation. One possible explanation might be the fact that FLC are not only produced and secreted within the IgG producing plasma cells but during all sorts of Ig production processes. Furthermore this quantitative method is independent of any mechanisms of clonal selection, that might be a more pronounced process in the later stages of CIS or MS. That could explain the significantly lower sensitivity of OB determination in CIS cohorts compared to results in MS patients. The mean KFLC-index of our MS group is significantly higher compared to our CIS group. An ongoing and increasing plasma cell activity in the MS disease course could explain this observation. None the less, the underlying reason for the immunological phenomenon of mainly KFLC production in the CSF in both MS and CIS patients remains to be established.

FLC quantification measures intrathecal plasma cell activity with high sensitivity and specificity outperforming IgG-index calculation. It is a fast, automated laboratory method that is easily integrated in routine diagnostics. The current study confirms reliable measurement of low KFLC concentrations that were regularly found in CSF. Results from these cohorts suggest the implementation of KFLC determination as a screening method in CSF analysis before further investigation for a possible intrathecal immune response, especially where MS or CIS is suspected.

FLC are small molecules (22 kD) that can accumulate in the CSF not only because of an intrathecal inflammatory process but also due to an altered blood-CSF barrier. In a former study [15] we demonstrated slightly elevated FLC levels in CSF in patients with GBS (where the detection of a blood-CSF barrier dysfunction is of high diagnostic relevance). We found raised FLC indices in patients who suffered from meningitis/encephalitis and whom had already reconstituted the blood-CSF barrier. When interpreting CSF results one has to be aware that a moderate elevation of both sorts of FLC in CSF, and the corresponding indices, can also be an indication of former blood-CSF barrier damage.

MS and CIS present with highly variable disease courses and to clearly verify a possible prognostic marker large cohorts of patients should be reviewed over decades of follow up. Several former studies [27]–[29] describe a correlation of elevated KFLC values with a worse MS conversion-rate and progression in comparison to patients with normal intrathecal KFLC levels. Attaching great importance to homogeneity we could investigate 58 MS and CIS patients for a possible prognostic value of the extent of KFLC-index elevation. Despite the variations in natural disease course the homogenous MS and CIS cohorts with moderately and highly elevated KFLC-index values show a very similar disease progression for the period under review (mean 55 months). Using an arbitrary cut-off we can conclude that in this study the absolute value of an elevated KFLC-index has no prognostic significance on MS or CIS disease course. Furthermore in this study there is no significant difference between the KFLC-index values of stable CIS patients and those suffering a conversion to MS. This must not be seen as a contradiction to the former reported observations that normal CSF results may be combined with a better prognosis in CIS and MS patients. However, normal CSF results, especially normal KFLC-index values are a rare finding in MS and CIS and the corresponding cohorts were too small for statistical analyses in our study. Whilst there is no overt role for the extent of elevation of KFLC-index in prognosis the clearly delineated raised KFLC-index levels shown here are of indisputable diagnostic relevance in MS and CIS.
